# Advanced Lung Cancer Inflammation Index Predicts Survival Outcomes of Patients With Oral Cavity Cancer Following Curative Surgery

**DOI:** 10.3389/fonc.2021.609314

**Published:** 2021-09-30

**Authors:** Yao-Te Tsai, Cheng-Ming Hsu, Geng-He Chang, Ming-Shao Tsai, Yi-Chan Lee, Ethan I. Huang, Chia-Hsuan Lai, Ku-Hao Fang

**Affiliations:** ^1^ Department of Otolaryngology—Head and Neck Surgery, Chang Gung Memorial Hospital, Chiayi, Taiwan; ^2^ Department of Medicine, College of Medicine, Chang Gung University, Taoyuan, Taiwan; ^3^ Department of Otolaryngology—Head and Neck Surgery, Chang Gung Memorial Hospital, Keelung, Taiwan; ^4^ Department of Radiation Oncology, Chang Gung Memorial Hospital, Chiayi, Taiwan; ^5^ Department of Otolaryngology—Head and Neck Surgery, Chang Gung Memorial Hospital, Taoyuan, Taiwan

**Keywords:** advanced lung cancer inflammation index, nomogram, biomarker, oral cavity squamous cell carcinoma (OSCC), overall survival, disease-free survival

## Abstract

**Aim:**

The aim of our study was to investigate the prognostic value of preoperative advanced lung cancer inflammation index (ALI) and to establish prognostic nomograms for the prediction of survival outcomes in patients with oral cavity squamous cell carcinoma (OSCC).

**Materials and Methods:**

A total of 372 patients who received primary curative surgery for OSCC during 2008–2017 at a tertiary referral center were enrolled. We used the receiver operating characteristic curve to determine the optimal cutoff point of ALI. Through a Cox proportional hazards model and Kaplan–Meier analysis, we elucidated the ALI–overall survival (OS) and ALI–disease-free survival (DFS) associations. Prognostic nomograms based on ALI and the results of multivariate analysis were created to predict the OS and DFS. We used the concordance indices (C-indices) and calibration plots to assess the discriminatory and predictive ability.

**Results:**

The results revealed that the ALI cutoff was 33.6, and 105 and 267 patients had ALI values of <33.6 and ≥33.6, respectively. ALI < 33.6 significantly indicated lower OS (44.0% vs. 80.1%, *p* < 0.001) and DFS (33.6% vs. 62.8%; *p* < 0.001). In multivariate analysis, ALI < 33.6 was independently associated with poor OS and DFS (both *p* < 0.001). The C-indices of established nomograms were 0.773 and 0.674 for OS and DFS, respectively; moreover, the calibration plots revealed good consistency between nomogram-predicted and actual observed OS and DFS.

**Conclusion:**

ALI is a promising prognostic biomarker in patients undergoing primary surgery for OSCC; moreover, ALI-based nomograms may be a useful prognostic tool for individualized OS and DFS estimations.

## Introduction

Squamous cell carcinoma (SCC) that inherently affects the head and neck is ranked sixth among the most common types of cancer worldwide, with oral cavity SCC (OSCC) representing a majority of the cases (1). In Taiwan, OSCC incidence continues to increase even though betel quid use has declined recently; possible reasons for the mentioned increase are the longstanding carcinogenic effects engendered by betel quid use and the damaging influence induced by chronic inflammation, cigarette smoking, and alcohol consumption (2, 3). Currently, the main therapeutic modalities for OSCC include primary curative surgery followed by adjuvant therapy (if indicated) and the definitive chemoradiotherapy (4, 5). Despite the advances in diagnostic modalities and modern multidisciplinary treatments over the past two decades, no significant improvement has been noted in OSCC survival rates; moreover, some patients still experience treatment failure due to locoregional recurrence or distant metastasis (4, 6).

Systemic inflammation and malnutrition are responsible for cancer growth, tumorigenesis, and metastasis, and these can be assessed by routine laboratory examinations at the time of diagnosis (7, 8); therefore, various nutrition/inflammation-based biomarkers, such as C-reactive protein–albumin ratio (CAR), platelet-to-lymphocyte ratio (PLR), neutrophil-to-lymphocyte ratio (NLR), and albumin–globulin ratio (AGR), are used for early estimation of head and neck cancer (HNC) prognosis (9–12). In 2013, Jafri et al. first combined NLR, serum albumin level, and body mass index (BMI) into a unified advanced lung cancer inflammation index (ALI) and demonstrated its prognostic value in patients who had been diagnosed as having metastatic non–small-cell lung cancer (NSCLC) (13). Subsequently, ALI was found to be useful for predicting survival outcomes in various cancers (14–16), including large B-cell lymphoma (15), small-cell lung cancer (SCLC) (17), NSCLC (18), esophageal SCC (19), HNC (20), and colorectal cancer (CRC) (21). Jank et al. first reported that a low ALI was in association with poor survival of patients with HNC (20). However, they enrolled only 21 (22.6%) patients with OSCC and did not include some substantial prognostic factors of OSCC, such as depth of invasion (DOI) (22) and extranodal extension (ENE) (23), in their survival analysis.

We speculated that ALI could be a prognostic index for OSCC; however, to apply ALI as a prognostic biomarker for OSCC, a comprehensive evaluation is required owing to the lack of relevant robust evidence. Moreover, the use of a biomarker-based nomogram—in addition to the traditional, tumor-factor–based tumor–node–metastasis (TNM) staging system—may facilitate prognostic stratification and individualized treatment planning in patients with OSCC (24, 25). However, thus far, data have not indicated the prognostic value of nomograms based on preoperative ALI in predicting disease-free survival (DFS) as well as overall survival (OS) in patients with OSCC. Herein, we determined whether ALI is correlated with DFS as well as OS in such patients receiving primary surgery. Furthermore, for predicting individualized 3- and 5-year DFS and OS in patients with OSCC, we established nomograms integrating ALI and independent prognosticators determined through multivariate analysis.

## Materials and Methods

### Study Design and Patient Population

In our single-center observational cohort research, we retrospectively drew our study population from an OSCC cohort who underwent curative surgery as a first-line treatment followed by adjuvant therapy if indicated at Chang Gung Memorial Hospital’s Department of Otolaryngology—Head and Neck Surgery over the period spanning from January 2008 to December 2017. The data analysis was conducted from April to July 2020. We executed patient selection on the basis of the following inclusion criteria: (1) being aged > 18 years, (2) having received a pathological diagnosis of invasive OSCC, and (3) having undergone primary surgery for OSCC in our hospital. By contrast, the exclusion criteria were as follows: (1) being diagnosed as having inoperable cancer or having a condition that constituted a contraindication for surgery, (2) having undergone neoadjuvant treatment before curative surgery, (3) having second primary cancer or distant metastasis when OSCC was diagnosed, (4) having a history of malignancy or hematologic disease, (5) having blood test results and the clinical symptoms and signs indicated severe infection status, and (6) having missing preoperative or follow-up data. Of the 391 eligible patients, we excluded 19 with missing data on variable of interest. Finally, 372 patients were analyzed further.

As shown in **Table 1**, we recorded each patient’s baseline and outcome data, including gender; age at diagnosis; primary tumor location; personal habits; *American Joint Committee on Cancer (AJCC) Staging Manual (Seventh Edition)*–based cancer stage; DOI; cancer cell differentiation; perineural invasion (PNI) status; ENE; nearest surgical margin; presence and types of adjuvant therapy; and underlying comorbidities based on the Charlson comorbidity index (CCI) (26), laboratory test results, and survival duration through electronic patient charts review.

**Table 1:** Baseline clinicopathological characteristics of patients with oral cavity squamous cell carcinoma (*n* = 372).

Variable	Characteristics
Age (years)	
<65	267 (71.8%)
≥65	105 (28.2%)
Gender	
Men	336 (90.3%)
Women	36 (9.7%)
BMI	
<23.1	140 (37.6%)
≥23.1	232 (62.4%)
Tumor site	
Buccal mucosa	120 (32.3%)
Tongue	143 (38.4%)
Others	109 (29.3%)
Personal Habits	
Cigarette smoking	305 (82.0%)
Alcohol consumption	248 (66.7%)
Betel nut chewing	292 (78.5%)
Overall stage	
I	86 (23.1%)
II	79 (21.2%)
III	44 (11.8%)
IV	163 (43.8%)
pT classification	
T1	106 (28.5%)
T2	115 (30.9%)
T3	23 (6.2%)
T4	128 (34.4)
pN classification	
N0	252 (67.7%)
N1	39 (10.5%)
N2	77 (20.7%)
N3	4 (1.1%)
PNI	91 (24.5%)
ENE	72 (19.4%)
Cell differentiation	
W−D/M−D	331 (89.0%)
P−D	41 (11.0%)
Surgical margin	
≥5 mm	272 (73.1%)
<5 mm	100 (26.9%)
DOI ≥ 10 mm	170 (45.7%)
Adjuvant therapy	
Absent	188 (50.5%)
Radiotherapy	49 (13.2%)
Chemo-radiotherapy	135 (36.3%)
CCI	
0	199 (53.5%)
1	144 (30.6%)
≥2	59 (15.9%)
Albumin (g/dl), mean ± SD	8.14 ± 2.69
WBC (×10^3^ μl^−1^), mean ± SD	8.14 ± 2.69
Neutrophil (×10^3^ μl^−1^), mean ± SD	5.28 ± 2.33
Lymphocyte (×10^3^ μl^−1^), mean ± SD	2.13 ± 0.71
ALI, mean ± SD	50.95 ± 28.14
NLR, mean ± SD	2.80 ± 1.72

ALI, advanced lung cancer inflammation; BMI, body mass index; CCI, Charlson comorbidity index; DOI, depth of invasion; ENE, extranodal extension; M−D, moderately differentiated squamous cell carcinoma; NLR, neutrophil-to-lymphocyte ratio; P−D, poorly differentiated squamous cell carcinoma; PNI, perineural invasion; SD, standard deviation; WBC, white blood cell; W−D, well-differentiated squamous cell carcinoma.

To explore the association between nutrition/inflammation indices and survival outcomes, the patients’ blood samples, height, and weight were routinely measured within a week preoperatively. We determined complete blood count and differential leukocyte count on an SE-9000 automated hematology analyzer (Sysmex, Kobe, Japan) and assayed serum albumin levels on a Cobas 8000 automated biochemistry analyzer (Roche Hitachi, Rotkreuz, Switzerland) according to the manufacturer’s instructions. BMI was derived using its established derivation formula: body weight (kg)/height squared (m^2^). The preoperative NLR was derived as follows: peripheral blood absolute neutrophil count divided by absolute lymphocyte count. In addition, ALI was derived using the following definition: [BMI (kg/m^2^) × serum albumin (g/dl)]/NLR (13).

### Treatment

All patients received primary curative surgery: wide excision of the primary tumor with unilateral or bilateral neck dissection. Intraoperative frozen section for surgical margin control was applied, and the surgical defects were immediately reconstructed by a plastic surgeon by using a free, pedicled, or local flap. Postoperative adjuvant therapy was applied on the basis of our institutional guidelines: it was administered within 6 weeks postoperatively if indicated. In brief, patients diagnosed as having a pathologic T4 disease and one metastatic lymph node are provided adjuvant radiotherapy, whereas those diagnosed as having positive surgical margins, ENE, or multiple metastatic lymphadenopathies are administered adjuvant concurrent chemoradiotherapy. Here, 66 Gy was the total adjuvant radiation dose delivered to the tumor site, with 2 Gy being provided over 5 days per week. For concurrent chemotherapy, the chemotherapy regimen consisted of intravenous cisplatin 40 mg/m^2^ weekly or 100 mg/m^2^ triweekly, depending on the oncologist’s judgment and patient’s preference. More detailed adjuvant therapy guidelines in our institute have been reported by Lin et al. (27).

### Follow-Up

The included study patients were all followed up at the outpatient clinic every 2, 3, and 6 months during the first, second, and third years after surgery, respectively. At every follow-up, the patients underwent complete physical examination, routine laboratory testing, and flexible endoscopy. Moreover, during the follow-up period, we executed magnetic resonance imaging or computed tomography at 6-month intervals during the first 2 years and annually thereafter. In this study, we defined OS as the period spanning from the date on which primary surgery was executed to the date on which the final follow-up was executed (i.e., December 31, 2019) or the date of censoring alive or death. We also defined DFS as the period spanning from the date on which primary surgery was executed to the date on which treatment failure (including locoregional recurrence, distant failure, censoring alive, or death) occurred or that on which the final follow-up was executed.

### Statistical Analysis

The categorical variables are shown as a number and percentage of the total, and we used the Kolmogorov–Smirnov test to evaluate the normality of continuous variables, which are represented as mean ± SD if normally distributed. To derive each study variable’s cutoff value, we executed receiver operating characteristic (ROC) curve analysis to estimate the optimal area under the ROC curve (AUC), sensitivity, and specificity for all-cause mortality prediction. Next, we stratified our patients and their OSCC tumor characteristics according to various clinicopathological features, followed by evaluation through the Mann–Whitney test (for continuous variables that are not normally distributed), the chi-square test (for categorical variables), and correlation testing, as appropriate. Kaplan–Meier curves for DFS and OS curves were plotted subsequently, and the survival differences were assessed for statistical significance using the log-rank test. After testing the proportionality for using Cox proportional hazard assumption, we identified independent risk factors of OS and DFS through the Cox proportional hazards model. In the univariable analysis, the factors were assessed using the log-rank test; those that reached statistical significance (*p* < 0.1) were included in the multivariable analysis using the Cox proportional hazard regression. In addition to ALI (i.e., the variable of interest) and its components, we considered variables previously identified as covariates influencing OSCC prognosis for our Cox analyses. These factors included age, gender, tumor location, overall stage (AJCC 7th edition), cancer cell differentiation, ENE, PNI, DOI (≥10 or <10 mm), surgical margin (≥5 or <5 mm), CCI (0, 1, or ≥2), and chemotherapy (yes or no). All aforementioned statistical analyses were executed using SPSS (version 21.0; SPSS Inc., Chicago, IL, USA), except the test for proportionality assumption, which was conducted with SAS version 9.4 (SAS Institute Inc). We also set the statistical significance level in this study at a two-tailed *p* of < 0.05.

To examine the advantage of using ALI as prognostic marker in clinical practice, we established nomograms integrating preoperative ALI, with the endpoints being 3- and 5-year OS and DFS, by employing the “rms” package of R (version 5.1-0; Vanderbilt University, Nashville, TN, USA) (28). For evaluating the nomograms’ predictive accuracy regarding 3- and 5-year DFS and OS, we calculated the corresponding concordance indices (C-indices) (28, 29); here, C-index values of 0.5 and 1.0 were considered to indicate random predictability and perfect matching, respectively (30, 31). Finally, the consistency of survival rates between the nomogram-predicted and observed values was determined by creating calibration plots for the nomograms.

## Results

### Baseline Characteristics

In total, 372 patients [336 (90.3%) men; mean (SD) age, 58.7 (11.1) years] were enrolled. **Table 1** lists the study cohort’s baseline demographic and clinicopathologic characteristics. The tongue was determined to constitute the most common primary tumor subsite (*n* = 143, 38.4%), followed by cheek mucosa (*n* = 120, 32.3%). A total of 207 patients (55.6%) had an advanced pathologic stage (stages III–IV) according to the AJCC staging system. History of cigarette smoking, alcohol consumption, and betel quid chewing were reported by 305 (82.1%), 248 (66.7%), and 292 (78.5%), respectively. In total, 188 (50.5%) patients underwent curative surgery alone, 135 (36.3%) underwent curative surgery followed by adjuvant chemoradiotherapy, and 49 (13.2%) underwent curative surgery with adjuvant radiotherapy. Comorbidities were recorded according to the CCI; 199 (53.5%), 144 (30.6%), and 59 (15.9%) patients had CCI scores of 0, 1, and ≥2 at the time of diagnosis, respectively. We determined the median follow-up duration for patients alive at the end of follow-up to be 58.5 (range: 2−126) months.

### ROC Curves of ALI and Its Components

**Figure 1:**
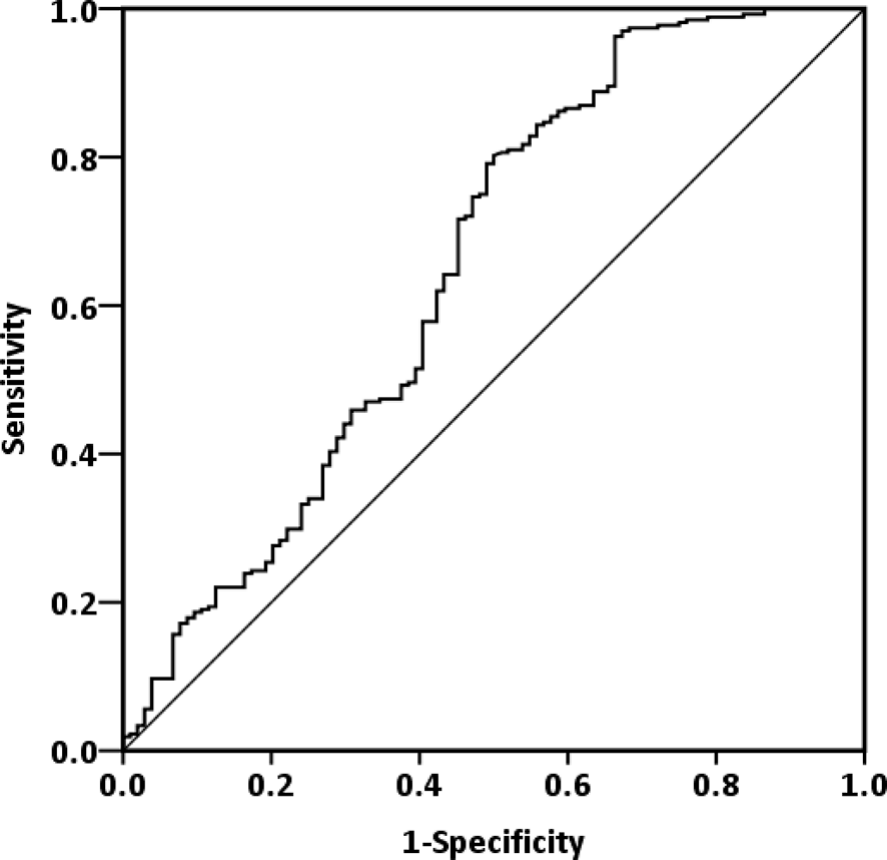
ROC curve of ALI of patients with operable OSCC.

By analyzing the ROC curve, we determined 33.6 as the optimal ALI cutoff [AUC: 0.693, 95% confidence interval (CI): 0.631−0.755, *p* < 0.001, **Figure 1**]. On the basis of this ALI cutoff, we separated the patients into the following groups: high-ALI (≥33.6, *n* = 267) and low-ALI (<33.6, *n* = 105) groups. ROC curves for OS associated with ALI and its component factors, including serum albumin level, BMI, neutrophil and lymphocyte counts, and the NLR, were also generated. By analyzing the ROC curves, we determined the optimal cutoff value to be 23.1 for BMI (*p* = 0.009), 4.51 for NLR (*p* = 0.001), and 4.21 for albumin (*p* < 0.001). The AUC for each factor is illustrated in **Table 2**. Nearly all component factors (except for neutrophil count, *p* = 0.094) could predict poor OS, but the AUC for ALI was significantly higher than those for the serum albumin level, neutrophil and lymphocyte counts, BMI, and NLR (all *p* < 0.001).

**Table 2:** Comparison of the area under the curve values of the ALI and its components.

Factor	AUC	95% CI	*p-*value	*p-*value^a^
Albumin	0.652	(0.584−0.719)	<0.001	<0.001
Neutrophils	0.556	(0.484−0.628)	0.094	<0.001
Lymphocyte	0.617	(0.552−0.682)	<0.001	<0.001
BMI	0.587	(0.519−0.654)	0.009	<0.001
NLR	0.614	(0.545−0.682)	0.001	<0.001
ALI	0.693	(0.631−0.755)	<0.001	–

ALI, advanced lung cancer inflammation index; AUC, area under the curve; BMI, body mass index; NLR, neutrophil-to-lymphocyte ratio.

a The AUC values between the advanced lung cancer inflammation index and other factors were compared using the Z-test method.

The Mann–Whitney U test (※ Z-test: albumin: −6.416; neutrophils: −8.739; lymphocyte: −8.837; BMI: −6.203; NLR: −13.758).

### Clinicopathological Features Based on the Cutoff of ALI

The relationship of ALI with demographic and clinicopathological factors is presented in **Table 3**. Compared with the high-ALI group, the low-ALI group had higher proportions of patients with low BMI (*p* < 0.001), advanced overall stage (III−IV, *p* < 0.001), T3–T4 and N1–N3 classifications (both *p* < 0.001), PNI (*p* = 0.001), ENE (*p* = 0.001), DOI ≥ 10 mm (*p* < 0.001), adjuvant therapy requirement (*p* < 0.001), CCI ≥ 2 (*p* = 0.014), high NLR (*p* < 0.001), low serum albumin levels (*p* < 0.001), and short survival (*p* < 0.001). Nevertheless, no such significant differences were noted for gender (*p* = 0.951), age (*p* = 0.926), cell differentiation (*p* = 0.207), or surgical margin (*p* = 0.750).

**Table 3:** Baseline clinicopathological characteristics according to the ALI.

Variable	Number of patients		*p-*value
	ALI < 33.6 (*n* = 105)	ALI ≥ 33.6 (*n* = 267)	
Gender			0.951^a^
Men	95 (90.5%)	241 (90.3%)	
Women	10 (9.5%)	26 (9.7%)	
Age			0.926^a^
<65	75 (71.4%)	192 (71.9%)	
≥65	30 (28.6%)	75 (28.1%)	
BMI			<0.001^a^
<23.1	61 (58.1%)	79 (29.6%)	
≥23.1	44 (41.9%)	188 (70.4%)	
Overall stage			<0.001^a^
I–II	24 (22.9%)	141 (52.8%)	
III–IV	81 (77.1%)	126 (47.2%)	
pT classification			<0.001^a^
T1–T2	36 (34.3%)	185 (69.3%)	
T3–T4	69 (65.7%)	82 (30.7%)	
pN classification			
N0	60 (57.1%)	192 (71.9%)	<0.001^a^
N1–N3	45 (42.9%)	75 (28.1%)	
PNI			
Absent	67 (63.8%)	214 (80.1%)	0.001^a^
Present	38 (36.2%)	53 (19.9%)	
ENE			0.001^a^
Absent	73 (69.5%)	227 (85.0%)	
Present	32 (30.5%)	40 (15.0%)	
Cell differentiation			0.207^a^
W–D/M–D	90 (85.7%)	241 (90.3%)	
P–D	15 (14.3%)	26 (9.7%)	
Surgical margin			0.750^a^
≥5 mm	78 (74.3%)	194 (72.7%)	
<5 mm	27 (25.7%)	73 (27.3%)	
TD ≥ 10 mm			<0.001^a^
No	34 (32.4%)	168 (62.9%)	
Yes	71 (67.6%)	99 (37.1%)	
Adjuvant therapy			<0.001^a^
Absent	34 (32.4%)	154 (57.7%)	
RT	15 (14.3%)	34 (12.7%)	
CCRT	56 (53.3%)	79 (29.6%)	
CCI			0.014^a^
0	53 (50.4%)	146 (54.7%)	
1	26 (24.8%)	88 (32.9%)	
≥2	26 (24.8%)	33 (12.4%)	
NLR, mean ± SD	4.76 ± 1.98	2.03 ± 0.68	<0.001^b^
Albumin(g/dl), mean ± SD	4.03 ± 0.69	4.47 ± 0.49	<0.001^b^
WBC (×10^3^ μl^−1^), mean ± SD	9.56 ± 3.55	7.58 ± 2.00	<0.001^b^
Neutrophil (×10^3^ μl^−1^), mean ± SD	7.16 ± 2.92	4.54 ± 1.52	<0.001^b^
Lymphocyte (×10^3^ μl^−1^), mean ± SD	1.61 ± 0.58	2.34 ± 0.65	<0.001^b^
Survival in months, mean ± SD	35.11 ± 27.08	54.16 ± 31.55	<0.001^b^

ALI, advanced lung cancer inflammation; BMI, body mass index; CCI, Charlson comorbidity index; CCRT, concurrent chemoradiotherapy; CI, confidence interval; DOI, depth of invasion; ENE, extranodal extension; M−D, moderately differentiated squamous cell carcinoma; NLR, neutrophil-to-lymphocyte ratio; P−D, poorly differentiated squamous cell carcinoma; PNI, perineural invasion; RT, radiotherapy; SD, standard deviation; WBC, white blood cell count; W−D, well differentiated squamous cell carcinoma.

a The chi-square test.

b The Mann–Whitney U test (※ Z-test: NLR: −13.758; albumin: −6.416; WBC: −5.486; neutrophil: −8.739; lymphocyte: −8.837; survival in months: −5.342).

### Factors Associated With Poor OS and DFS

**Figure 2:**
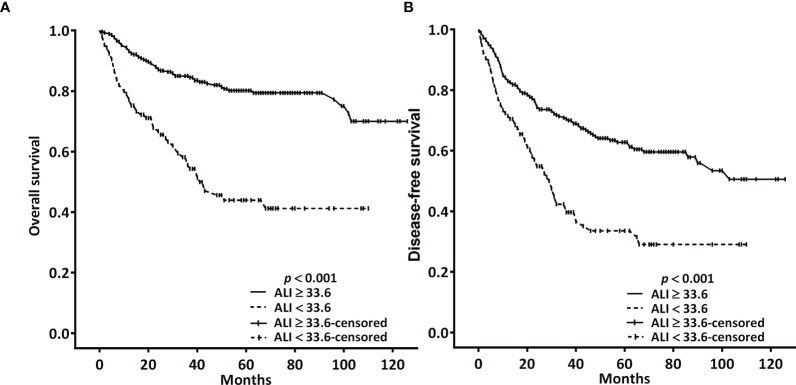
Kaplan–Meier curves for **(A)** OS and **(B)** DFS of OSCC patients with ALI of ≥33.6 and <33.6.

The low-ALI group had a shorter median OS period than the high-ALI group did (3.4 vs. 8.6 years). According to the Kaplan–Meier survival curve, the 5-year OS rates in the high- and low-ALI groups were 80.1% and 44.0%, respectively, signifying a remarkable difference, as revealed by the log-rank test (*p* < 0.001; **Figure 2A**). Moreover, the low-ALI group had a shorter median DFS period than did the high-ALI group (2.4 vs. 7.9 years). According to the Kaplan–Meier survival curve, the 5-year DFS rates in the high- and low-ALI groups were 62.8% and 33.6%, respectively, signifying a substantial difference, as demonstrated by the log-rank test (*p* < 0.001; **Figure 2B**).

**Table 4** presents the results of the univariate analysis for each factor with regard to OS and DFS predictions. We noted significant correlations of OS and DFS with overall stage, ENE, cancer cell differentiation, DOI, chemotherapy, serum albumin level, NLR, and ALI. Because NLR and serum albumin level are component factors of ALI, we performed a separate multivariate analysis to prevent high collinearity; the results indicated advanced overall stage, the presence of ENE, poor cancer cell differentiation, serum albumin level of < 4.21, NLR of ≥ 4.51, and ALI of <33.6 to be independent prognostic factors for poor OS and DFS (**Table 5**).

**Table 4:** Univariate analysis of poor prognostic factors for OS and DFS in OSCC patients.

Variable	OS		DFS	
	HR (95% CI)	*p-*value	HR (95% CI)	*p-*value
Gender				
Women	Reference		Reference	
Men	1.217 (0.614–2.412)	0.574	1.271 (0.637–2.536)	0.496
Age (years)				
<65	Reference		Reference	
≥65	1.240 (0.823–1.868)	0.303	0.743 (0.453–1.216)	0.237
BMI				
<23.1	Reference		Reference	
≥23.1	1.635 (0.912–2.402)	0.062	1.321 (0.968–1.803)	0.079
Overall stage				
I	Reference		Reference	
II	1.513 (0.609–3.763)	0.373	0.764 (0.434–1.346)	0.352
III	2.783 (1.118–6.928)	0.028	1.259 (0.696–2.280)	0.446
IV	6.779 (3.259–14.102)	<0.001	2.407 (1.576–3.675)	<0.001
PNI				
Absent	Reference		Reference	
Present	1.513 (0.609–3.763)	0.373	1.049 (0.651–1.691)	0.844
ENE				
Absent	Reference		Reference	
Present	3.955 (2.663–5.873)	<0.001	1.803 (1.116–2.912)	0.016
Cell differentiation				
W–D/M–D	Reference		Reference	
P–D	2.954 (1.840–4.743)	<0.001	1.910 (1.185–3.078)	0.008
DOI ≥ 10 mm				
No	Reference		Reference	
Yes	2.243 (1.514–3.324)	<0.001	1.363 (0.973–1.909)	0.072
Tumor subsites				
Tongue	Reference		Reference	
Buccal mucosa	1.151 (0.722–1.833)	0.555	1.167 (0.798–1.706)	0.425
Other	1.160 (0.723–1.861)	0.537	1.352 (0.928–1.968)	0.116
Surgical margin				
≥5 mm	Reference		Reference	
<5 mm	1.406 (0.935–2.114)	0.101	1.308 (0.939–1.820)	0.112
CCI				
0	Reference		Reference	
1	1.245 (0.790–1.960)	0.345	0.853 (0.591–1.233)	0.398
≥2	1.976 (0.922–3.196)	0.076	1.213 (0.805–1.828)	0.356
Chemotherapy				
Yes	Reference		Reference	
No	0.301 (0.202–0.444)	<0.001	0.508 (0.372–0.693)	<0.001
Albumin				
<4.21	Reference		Reference	
≥4.21	3.551 (2.415–5.222)	<0.001	2.123 (1.549–2.910)	<0.001
ALI				
≥33.6	Reference		Reference	
<33.6	3.477 (2.359–5.126)	<0.001	2.067 (1.473–2.900)	<0.001
NLR				
<4.51	Reference		Reference	
≥4.51	4.845 (3.176–7.389)	<0.001	2.992 (2.057–4.351)	<0.001

ALI, advanced lung cancer inflammation; BMI, body mass index; CCI, Charlson comorbidity index; CI, confidence interval; DFS, disease-free survival; DOI, depth of invasion; ENE, extranodal extension; HR, hazard ratio; M–D, moderately differentiated squamous cell carcinoma; NLR, neutrophil-to-lymphocyte ratio; OS, overall survival; OSCC, oral cavity squamous cell carcinoma; P–D, poorly differentiated squamous cell carcinoma; PNI, perineural invasion; W–D, well-differentiated squamous cell carcinoma.

**Table 5:** Multivariate analysis of poor prognostic factors for OS and DFS in OSCC patients.

Variable	Albumin-NLR model		ALI model	
	HR (95% CI)	*p-*value	HR (95% CI)	*p-*value
**Overall survival**				
Overall stage				
I	Reference		Reference	
II	1.674 (0.663–4.227)	0.276	1.799 (0.712–4.547)	0.214
III	2.749 (1.068–7.075)	0.036	2.458 (1.152–6.344)	0.043
IV	3.990 (1.685–9.446)	0.002	4.153 (1.751–9.849)	0.001
ENE				
Absent	Reference		Reference	
Present	2.194 (1.397–3.446)	0.001	2.161 (1.372–3.404)	0.001
Cell differentiation				
W–D/M–D	Reference		Reference	
P–D	2.279 (1.375–3.778)	0.001	2.463 (1.495–4.060)	<0.001
DOI ≥ 10 mm				
No	Reference		Reference	
Yes	0.977 (0.606–1.575)	0.924	0.945 (0.593–1.507)	0.814
Chemotherapy				
Yes	Reference		Reference	
No	0.955 (0.563–1.620)	0.864	1.133 (0.675–1.899)	0.637
Albumin				
≥4.21	Reference			
<4.21	2.450 (1.621–3.703)	<0.001		
ALI				
≥33.6			Reference	
<33.6			2.519 (1.678–3.780)	<0.001
NLR				
<4.51	Reference			
≥4.51	2.384 (1.507–3.772)	<0.001		
**Disease-free survival**				
Overall stage				
I	Reference		Reference	
II	0.852 (0.475–1.526)	0.589	0.906 (0.507–1.621)	0.740
III	1.337 (0713–2.505)	0.365	1.312 (0.701–2.458)	0.396
IV	1.918 (1.095–3.361)	0.023	2.043 (1.165–3.584)	0.013
ENE				
Absent	Reference		Reference	
Present	1.980 (1.336–2.935)	0.001	2.018 (1.358–2.991)	0.001
Cell differentiation				
W–D/M–D	Reference		Reference	
P–D	1.941 (1.255–3.016)	0.003	1.958 (1.272–3.013)	0.002
DOI ≥ 10 mm				
No	Reference		Reference	
Yes	0.869 (0.590–1.281)	0.478	0.832 (0.565–1.226)	0.353
Chemotherapy				
Yes	Reference		Reference	
No	0.853 (0.556–1.309)	0.467	0.909 (0.594–1.391)	0.660
Albumin				
≥4.21	Reference			
<4.21	1.642 (1.170–2.304)	0.004		
ALI				
≥33.6			Reference	
<33.6			2.016 (1.353–3.011)	<0.001
NLR				
<4.51	Reference			
≥4.51	1.987 (1.324–2.983)	0.001		

ALI, advanced lung cancer inflammation; BMI, body mass index; CI, confidence interval; DFS, disease-free survival; DOI, depth of invasion; ENE, extranodal extension; HR, hazard ratio; M–D, moderately differentiated squamous cell carcinoma; NLR, neutrophil-to-lymphocyte ratio; OS, overall survival; OSCC, oral cavity squamous cell carcinoma; P–D, poorly differentiated squamous cell carcinoma; PNI, perineural invasion; W–D, well differentiated squamous cell carcinoma.

### Subgroup Analysis for Discriminatory Ability of ALI

**Figure 3:**
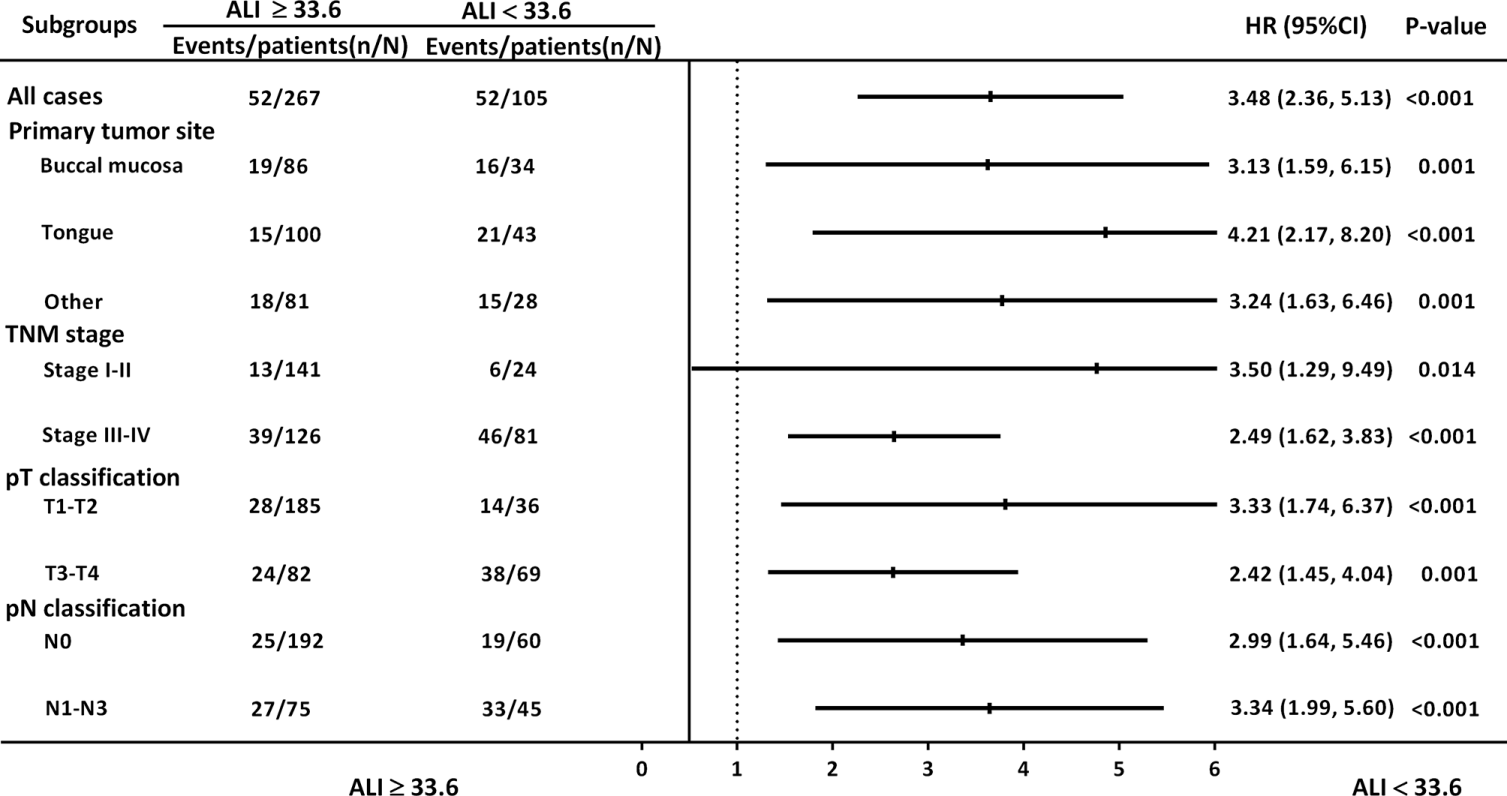
HRs for ALI in subgroup analysis, stratified by primary tumor site, overall stage, and pT and pN classification.

**Figure 3** illustrates the subgroup analysis results: ALI was associated with OS with respect to different primary tumor sites [hazard ratio (HR): 3.13, 95% CI: 1.59–6.15, *p* = 0.001 for buccal cancer; HR: 4.21, 95% CI: 2.17–8.20, *p* < 0.001 for tongue cancer], early stage (I–II) disease (HR: 3.50, 95% CI: 1.29–9.49, *p* = 0.014), advanced-stage (III–IV) disease (HR: 2.49, 95% CI: 1.62–3.83, *p* < 0.001), early pT classification (HR: 3.33, 95% CI: 1.74–6.37, *p* < 0.001), late pT classification (HR: 2.42, 95% CI: 1.45–4.04, *p* = 0.001), and different pN classifications (HR: 2.99, 95% CI: 1.64–5.46, *p* < 0.001 for N0; HR: 3.34, 95% CI: 1.99–5.60, *p* < 0.001 for N1–N3).

### Nomograms for Survival Prediction in OSCC

**Figure 4:**
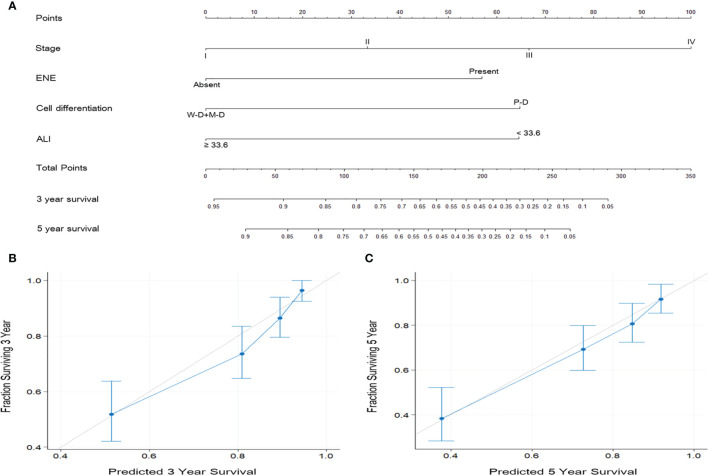
**(A)** Nomogram based on ALI and independent prognostic factors for OS prediction. Each parameter is included as a line segment on the nomogram, and the points on the line segment indicate the degree of risk contributed by this parameter. Addition of the points for all parameters yields the total points corresponding to the 3- and 5-year OS rates for the individual patient. **(B, C)** Calibration plots of the nomogram for **(B)** 3-year and **(C)** 5-year OS prediction. The light gray 45° line indicates the ideal prediction, and the blue line represents the value predicted by the nomogram model.

**Figure 5:**
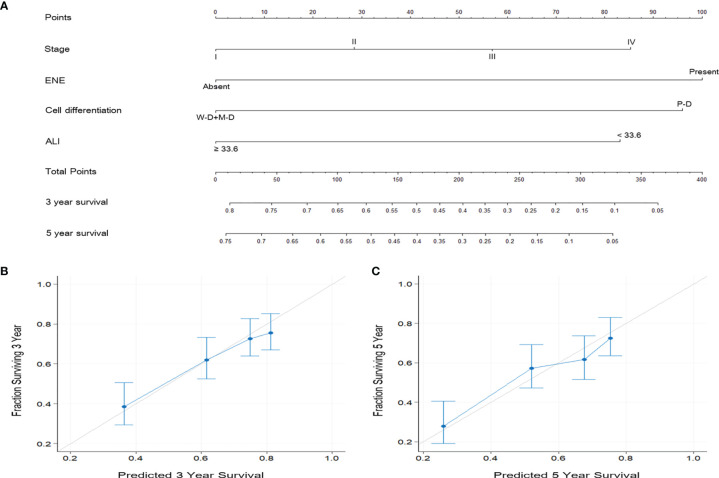
**(A)** Nomogram based on ALI and independent prognostic factors for DFS prediction. **(B, C)** Calibration plots of the nomogram for **(B)** 3-year and **(C)** 5-year DFS prediction. The predicted values were in good agreement with the observed values.

According to the multivariate analysis results, independent prognostic factors such as overall stage, ENE, cancer cell differentiation, and ALI were incorporated to establish the prognostic nomograms for the prediction of OS (**Figure 4A**) and DFS (**Figure 5A**) in patients with OSCC. The AUC of the nomograms was 0.81 (sensitivity: 70.3%, specificity: 76.1%) for OS prediction and 0.72 (sensitivity: 66.5%, specificity: 67.3%) for DFS prediction. The C-index (95% CI) of the nomograms was 0.773 (0.744–0.803) for OS and 0.674 (0.651–0.698) for DFS, suggesting the established nomograms to exhibit acceptable to good predictive accuracy and discrimination performance. We further used calibration plots to evaluate the consistency between the observed values and values predicted by the nomogram models. The calibration plots for the 3-year (**Figure 4B**) and 5-year (**Figure 4C**) OS rates predicted by the established nomograms were noted to be quite close to the 45° standard line, indicating that the nomograms had a good degree of calibration. Similarly, the calibration plots demonstrated that the 3- and 5-year DFS rates (**Figures 5B, C**, respectively) predicted by the nomograms were in good agreement with the actual observed values.

## Discussion

The conventional AJCC and UICC staging systems, covering the tumor extent and status of neck nodal metastasis and distant metastasis, are currently the most widely used references for prognosis prediction and cancer treatment. Nevertheless, the AJCC staging system focuses only on tumor factors and lacks certain clinicopathological or demographic factors that potentially influence survival outcomes. Because the NLR reflects the systemic immunoinflammatory response (32) and because cachexia—a result of chronic systemic inflammation—is possibly reflected by BMI and serum albumin level (33), pretreatment ALI may reflect the underlying equilibrium of patient’s nutritional status and systemic inflammation and can predict survival outcomes for various malignant tumors (14–21) with superior discriminatory ability than the index based solely on inflammation (16, 34). In the present study, through a retrospective analysis of 372 patients’ clinicopathological features and survival outcomes, the prognostic ability of ALI in patients who underwent surgical treatment for OSCC was determined. According to the ROC curve analysis results, ALI demonstrated the highest AUC for BMI, albumin level, NLR, and neutrophil and lymphocyte counts, suggesting that ALI had a higher discriminatory ability for OS prediction than did its component facts because of its combination of body stature, host nutrition condition, and systemic inflammation patterns. Moreover, a low ALI (<33.6) was significantly associated with advanced-stage disease, advanced pT and pN classifications, PNI, ENE, need for adjuvant therapy, DOI >10 mm, and short survival period, indicating the importance of pretreatment screening for inflammatory status and malnutrition with regard to disease aggressiveness and prognosis. Our Kaplan–Meier analysis and log-rank test revealed that a low ALI was significantly associated with worse 5-year OS and DFS rates. Because ALI was calculated using BMI, the NLR, and albumin level, we conducted separate multivariate analyses to avoid the collinearity, and the results demonstrate that the association of advanced overall stage, presence of ENE, poor cell differentiation, low albumin, high NLR, and low ALI with poor DFS and OS was strong. These findings were consistent with previous studies, as the pretreatment serum albumin levels and NLR predicts survival outcomes in patients with HNC (35, 36).

Notably, the HRs of low ALI in predicting poor OS and DFS were 2.519 and 2.016, respectively, both of which were higher than that of albumin and NLR. In addition, our subgroup analysis revealed that the prognostic value of ALI for OS in patients with OSCC was consistent across different oral subsites, early and advanced-stage disease, and early and late T and N classifications. This observation not only highlights the general applicability of ALI in both metastatic lung cancer and OSCC but also suggests the consistent prognostic value of ALI in OSCC. In addition, we further integrated preoperative ALI and independent prognostic factors to derive prognostic nomograms for OS and DFS prediction in OSCC. The C-indices of the established nomograms were determined to be higher than that of the AJCC staging system–based nomogram model (0.773 vs. 0.699 for OS and 0.674 vs. 0.628 for DFS). After verifying the discriminatory ability, we created calibration plots for 3- and 5-year OS and DFS estimates and found that the nomograms had a good degree of calibration. Taken together, these findings suggest that preoperative ALI has potential value as a prognostic biomarker in patients with OSCC.

A low ALI has been revealed by previous studies to be associated with poor prognosis in various cancer types (14–16) because ALI integrates the factors essential in host immune, nutritional, and systemic inflammation statuses—namely, serum albumin level, BMI, and NLR. However, the definite mechanisms underlying the contribution of ALI to the survival outcomes of patients with OSCC have not been delineated yet. Thus far, serum albumin level, BMI, and NLR have been revealed to be significantly and independently associated with poorer survival outcomes in cancers, including OSCC. A meta-analysis of 25 studies including 6,847 patients with SCC of the head and neck concluded that a high pretreatment NLR strongly predicts poor progression-free survival, DFS, OS, and cancer-specific survival (12); a partial explanation for this may be that angiogenesis and extracellular matrix remodeling may potentially be promoted by cytokines and chemokines produced by neutrophils in order to provide a favorable microenvironment for cancer growth (37). Moreover, lymphocytes have a vital role in antitumor immunity; they destroy malignant cells by identifying tumor cell antigens (38). The relationship between malnutrition (reflected by BMI and serum albumin level) and cancer prognosis has also been extensively assessed for various cancers (39). Takenaka et al. indicated that a low pretreatment BMI is a prognostic factor for poor OS in patients with SCC of the head and neck who underwent definitive radiotherapy or chemoradiotherapy (40); this finding may be attributed to the cancer-related oxidative stress, chronic wasting, and relatively high protein metabolism caused by cancer cachexia (41).

Serum albumin may also serve as a surrogate for nutritional status. Lim et al. conducted a prospective cohort study on 338 patients with SCC of the head and neck who received definitive treatment and reported that pretreatment hypoalbuminemia constituted an independent risk factor for poor DFS, CSS, and OS, which may be explained by the fact that inflammation mediated by cancer may reduce serum albumin levels by expanding microvasculature permeability and the transcapillary passage of serum albumin and controlling albumin synthesis in the liver through cytokine mediation (36). Considering these results and the previous observations by Jank et al. in patients with HNC (20), we believed that a low ALI, which reflects a host’s poor immuno-nutritional status and overexpressed systemic inflammation overall, predicted worsened prognosis in patients with surgically treated OSCC. However, the definite mechanism underlying the ALI–survival outcome association in OSCC warrants further investigation.

In this study, ALI had the highest AUC for OS compared with its components. Because BMI and serum albumin levels are affected by various factors, such as cancer- or treatment-related malnutrition, body fluid volume changes, and hepatic insufficiency, considering BMI or albumin level alone may be insufficient for survival outcome prediction in patients with OSCC. Similarly, the NLR may be influenced by an indolent infection or chronic inflammation condition. Hence, ALI may provide a more comprehensive assessment with less measurement variability, making it a more stable indicator than BMI, serum albumin level, or NLR alone for the simultaneous demonstration of a patient’s immune, nutritional, and systemic inflammation status. BMI was also reported to be correlated with sarcopenia, a major component of cancer–cachexia syndrome and a negative predictor of prognosis of SCC of the head and neck (42). However, it may not be applicable for the precise interpretation of fat versus muscle composition (43). Some patients diagnosed as having sarcopenic obesity have low skeletal muscle mass but heavy body weight because of their high fat mass. Hence, Kim et al. used the L3 skeletal muscle index (SMI) measured through computed tomography to replace BMI and developed a modified ALI (14). Nevertheless, they found that the modified L3 SMI–based ALI had no additional prognostic value beyond the original BMI-based ALI in patients with SCLC and concluded that the original ALI was simple but robust for prognosis prediction. These findings confirm the relevance of our study results: ALI has high prognostic value in patients who are to undergo primary surgery for OSCC.

The AJCC staging system has always been an important reference with generalizability and applicability for cancer treatment and prognostic prediction globally. Nevertheless, it does not consider certain OSCC characteristics and demographic features that are frequently considered by clinicians when making treatment decisions. Nomograms are reliable and individualized prediction tools widely applied in oncology research and enable clinical physicians to conveniently execute practical assessments (44). Through the incorporation of diverse prognostic factors, nomograms can determine the probability of a clinical event (e.g., OS or DFS) and thus predict the prognosis of a single patient. Nomograms are necessary in the era of individualized oncological therapy, and several nomograms have been published as adjuncts in prognostic determination in different types of cancer, such as CRC (45), renal cancer (46), NSCLC (47), and gastric cancer (48). Regarding the HNC, the nomograms have also been constructed to estimate the risk of developing major surgical complications in OSCC patients (49) and predict the recurrence-free probability in patients with parotid cancer (50). As we determined after executing a literature review, the current study is the first to establish ALI-based nomograms; the main advantage of our established nomograms is their strong discriminatory ability in estimating individualized 3- and 5-year OS and DFS rates. Our multivariate analysis revealed advanced overall stage, presence of ENE, poor cell differentiation, and low ALI to be independent adverse predictors of OS and DFS in patients with OSCC; therefore, we constructed nomograms based on these independent prognostic factors with feasible results (C-index: 0.773 for OS and 0.674 for DFS). Calibration plots demonstrated high consistency between the OS and DFS predictions provided by the established nomograms and observed values. As illustrated in **Figures 4** and **5**, the calibration plots of the nomograms are quite close to the ideal 45° line with even distributions, suggesting that the incidence rates predicted by the nomograms were close to the actual observed incidence rates. These results confirm the high performance of the established ALI-based nomograms and verify that they can be used for the prediction of individualized 3- and 5-year OS and DFS rates in OSCC, possibly aiding surgeons in identifying patients who may benefit more from aggressive treatment and therefore influencing all aspects of cancer care, including the survival outcomes.

Although the ROC curve analysis revealed 33.6 to be the cutoff for ALI in our study, different ALI cutoff values have been adopted in other studies. Thus, the optimal threshold remains uncertain. In the first-ever study on ALI, Jafri et al. reported that the cutoff ALI value was 18, but they analyzed only 173 patients who were undergoing palliative chemotherapy for metastatic NSCLC (13). For patients with SCLC, Kim et al. (14) and He et al. (17) have reported cutoff values of 31.1 and 19.5, respectively. Moreover, Jank et al. indicated that ALI had prognostic value at a cutoff of 37.6 in 93 patients with HNC (20), similar to the cutoff value (i.e., 37.66) reported for resected NSCLC by Tomita et al. (18). Our analysis also provided a cutoff within the range of those reported previously. However, the lack of uniformity in the ALI cutoff values among the various cancers may impede the general applicability of pretreatment ALI in clinical practice; thus, further relevant investigation is warranted.

At present, the mainstay of treatment strategies should chiefly be based on the National Comprehensive Cancer Network (NCCN) guidelines, rather than inflammatory markers such as ALI. Of note, the indications of adjuvant therapy for patients with OSCC in our institute were not completely in line with the NCCN guidelines, and the results of their comparison have been reported by our colleague (27). However, our results reveal that preoperative ALI is a useful, convenient preoperative marker in patients with OSCC and that nomograms based on ALI may exhibit a high predictive accuracy for OS and DFS, both of which are meaningful findings. In general, in this study, the prognostic value of preoperative ALI was investigated by including a relatively large cohort of patients who underwent surgery for OSCC. We suggest that for convenience, preoperative ALI can be determined through routine blood test results and body stature measurements, and after surgery, nomograms can be derived by incorporating ALI and clinicopathological factors, thus developing a reliable and cost-effective tool for predicting OSCC prognosis and stratifying patients for suitable adjuvant therapy; this can ultimately facilitate the management of surgically treated OSCC in clinical settings. The current study’s limitations are as follows: First, our study design (single-institution and retrospective design) has inherent limitations that cannot be completed excluded, such as information bias. Second, our results could not be verified on an independent data set; thus, their external validity remains unconfirmed. The established nomogram may also suffer from over-optimism because it was developed and evaluated on the same database (51). Future large-scale, prospective cohort studies along with external validation are thus warranted.

## Conclusions

The preoperative ALI may be applicable as a prognostic biomarker in patients with operable OSCC. Here, a low preoperative ALI was found to be associated with aggressive clinicopathological characteristics; it was also an independent risk factor for poor OS and DFS. Nomograms incorporating ALI into the conventional AJCC staging system might provide accurate prognostic information regarding OS and DFS to clinicians, thus enabling them to optimize adjuvant therapy and administer personalized treatment. Given the convenience and cost-effectiveness of this biomarker, ALI is a highly favorable candidate for use in clinical and oncology research.

## Data Availability Statement

The raw data supporting the conclusions of this article will be made available by the authors, without undue reservation.

## Ethics Statement

Chang Gung Memorial Hospital’s Institutional Review Board ratified our executed study, which followed the tenets of the Declaration of Helsinki. Furthermore, the hospital’s board exempted our study from the established requirement of receiving patient informed consent (approval no. 202000657B0).

## Author Contributions

Y-TT and G-HC conceived, designed, and supervised the study. M-ST, Y-CL, and EH collected the data of patients and followed up. C-HL and K-HF analyzed the data. C-HL and G-HC provided technical assistance with the data analysis. Y-TT, K-HF, and C-MH wrote the manuscript. All authors contributed to the article and approved the submitted version.

## Funding

This work was supported by a grant (CMRPG6J0202) from Chang Gung Memorial Hospital, Taiwan.

## Conflict of Interest

The authors declare that the research was conducted in the absence of any commercial or financial relationships that could be construed as a potential conflict of interest.

## Publisher’s Note

All claims expressed in this article are solely those of the authors and do not necessarily represent those of their affiliated organizations, or those of the publisher, the editors and the reviewers. Any product that may be evaluated in this article, or claim that may be made by its manufacturer, is not guaranteed or endorsed by the publisher.
